# Structural decomposition of decadal climate prediction errors: A Bayesian approach

**DOI:** 10.1038/s41598-017-13144-2

**Published:** 2017-10-09

**Authors:** Davide Zanchettin, Carlo Gaetan, Maeregu Woldeyes Arisido, Kameswarrao Modali, Thomas Toniazzo, Noel Keenlyside, Angelo Rubino

**Affiliations:** 1 University Ca’Foscari of Venice, Dept. of Environmental Sciences, Informatics and Statistics, Via Torino 155, 30170 Mestre Venezia, Italy; 2 Max Planck Institute for Meteorology, Bundesstrasse 53, 20146 Hamburg, Germany; 3 Uni Research, Bjerknes Centre for Climate Research, Bergen, Norway; 4 Geophysical Institute, University of Bergen, Postboks 7803, 5020 Bergen, Norway

## Abstract

Decadal climate predictions use initialized coupled model simulations that are typically affected by a drift toward a biased climatology determined by systematic model errors. Model drifts thus reflect a fundamental source of uncertainty in decadal climate predictions. However, their analysis has so far relied on ad-hoc assessments of empirical and subjective character. Here, we define the climate model drift as a dynamical process rather than a descriptive diagnostic. A unified statistical Bayesian framework is proposed where a state-space model is used to decompose systematic decadal climate prediction errors into an initial drift, seasonally varying climatological biases and additional effects of co-varying climate processes. An application to tropical and south Atlantic sea-surface temperatures illustrates how the method allows to evaluate and elucidate dynamic interdependencies between drift, biases, hindcast residuals and background climate. Our approach thus offers a methodology for objective, quantitative and explanatory error estimation in climate predictions.

## Introduction

The multiannual forecast horizon inherent to decadal climate prediction requires that the complexity and uncertainties arising from the interaction between the climate response to external forcing and the evolution of internal modes of climate variability are accounted for^1,2^. To generate an ensemble of decadal forecasts, various procedures are applied in climate models to obtain sets of initial conditions based on the observed state of the climate system at a certain time^2^. The observed evolution of many geophysical parameters however resides outside the climatological envelope simulated by coupled climate models used in contemporary climate prediction systems, due to the presence of large systematic model biases with respect to observations^3–5^. These affect all of mean state, seasonal cycle and interannual internal variability. Decadal climate forecasts based on full-field initialization therefore unavoidably include a growing systematic error, which corresponds with the adjustment of the simulations from the assimilated state drawn from the observed climatology towards a state consistent with the biased climatology of the model^2^. This signal is commonly referred to as climate model drift^6^.

Model drifts and biases can result from the erroneous representation of oceanic and atmospheric processes in climate models^1,4,7–11^, but more generally they reflect our limited understanding of many of the interactions and feedbacks in the climate system and approximations and simplifications inherent to the numerical representation of climate processes (so-called parameterizations). Sea-surface temperature (SST) biases are often central in the characterization of climate model biases^3^. Typical examples of systematic model bias are the warm and weak upwelling systems at the eastern boundaries of the tropical oceans^12–14^. Among these, the severe warm SST bias in the southeastern tropical Atlantic has been especially studied^5,12,15–17^.

A multi-model analysis of SST drifts in this region highlighted that similar biases arise there in different models through a variety of locally as well as remotely growing error mechanisms^12^. The dynamical processes governing model drifts (or their numerical representation) are identical to those of the simulated climate itself, so that climate and error signals are indistinguishable by first principles. The fact that model biases and drifts often have a prominent seasonal character^11,16^ further increases the difficulty of evaluating, quantifying and understanding their impacts on the overall quality of simulated climates. Various techniques for drift mitigation have been proposed, such as anomaly initialization or flux correction^18^. Nonetheless, in decadal climate predictions model drifts remain most often regarded as mere biases, which are often corrected for by means of empirical techniques with varying complexity^19–22^, but rarely investigated in depth^23^.

A substantial improvement in our understanding of mechanisms contributing to the generation and propagation of systematic decadal climate prediction errors may be achieved by means of a process-oriented statistical characterization of their temporal and spatial complexity and a robust quantification of associated uncertainties. Here, we propose a structural decomposition analysis^24^ of systematic hindcast errors (i.e., systematic discrepancies between values from “retrospective predictions” and the corresponding observation) in an ensemble of decadal climate predictions. Structural decomposition refers to a process model in which temporal changes of an observable variable are attributed to the discriminant effects (or “driving forces”) underlying these changes. Accordingly, each time series of hindcast errors D_j_(*t*) in the ensemble is represented as the sum of a systematic component Δ(*t*), which captures the (systematic) portion of hindcast errors common to all realizations, and a non-systematic irregular component ε_j_(*t*), which is specific to the individual considered hindcast realization *j*. Here, Δ(*t*) is defined as a linear combination of systematic hindcast error δ(*t*) and systematic seasonal bias σ(*t*). In particular, δ(*t*) evolves as a stochastic trend with slope β(*t*): initially, when β(*t*) ≠ 0, δ(*t*) describes the drift; then, when β(*t*) ≈ 0, δ(*t*) corresponds to the climatological bias. Moreover, Δ(*t*) can incorporate the explanatory effects of a covariate X(*t*) representing local and/or remote processes and quantified by the associated stochastic regression coefficient γ(*t*) of Δ(*t*) on X(*t*). As discriminant effects are non-observable, a state-space model is built in which the state vector includes all unobserved elements and the transition matrix describes their individual dynamics (see the methods section for details).

The variances of the error terms in the state-space model are unknown parameters, and inference is performed following a Bayesian approach that specifies prior distributions for them. Therefore, our modeling strategy consists of three hierarchical levels (see the methods section for details): the data level, where observational errors, ε_j_(*t*), for all hindcasts are accounted for conditionally on the systematic component Δ(*t*); the process level, where the systematic components of the bias, δ(*t*) and σ(*t*), and its constituting discriminant effects, X(*t*), are represented by means of a stochastic model; and the parameter level, where prior knowledge about the distributions of model errors (via the parameters τ^2^) is formalized.

In this contribution, we choose to illustrate our hierarchical statistical framework and its value for a robust quantification of systematic model prediction errors and their associated uncertainty by means of a specific example. We discuss the diagnosed distinctive characteristics of the different error components and use them for a reliable identification of connections between drift dynamics and the physics of the simulated climate. This study thus focuses on SST errors in the Tropical and South Atlantic Ocean estimated for an ensemble of decadal hindcasts initialized from observations, with the MiKlip prototype system for decadal climate prediction^25^ (see Methods).

## Results

**Figure 1:**
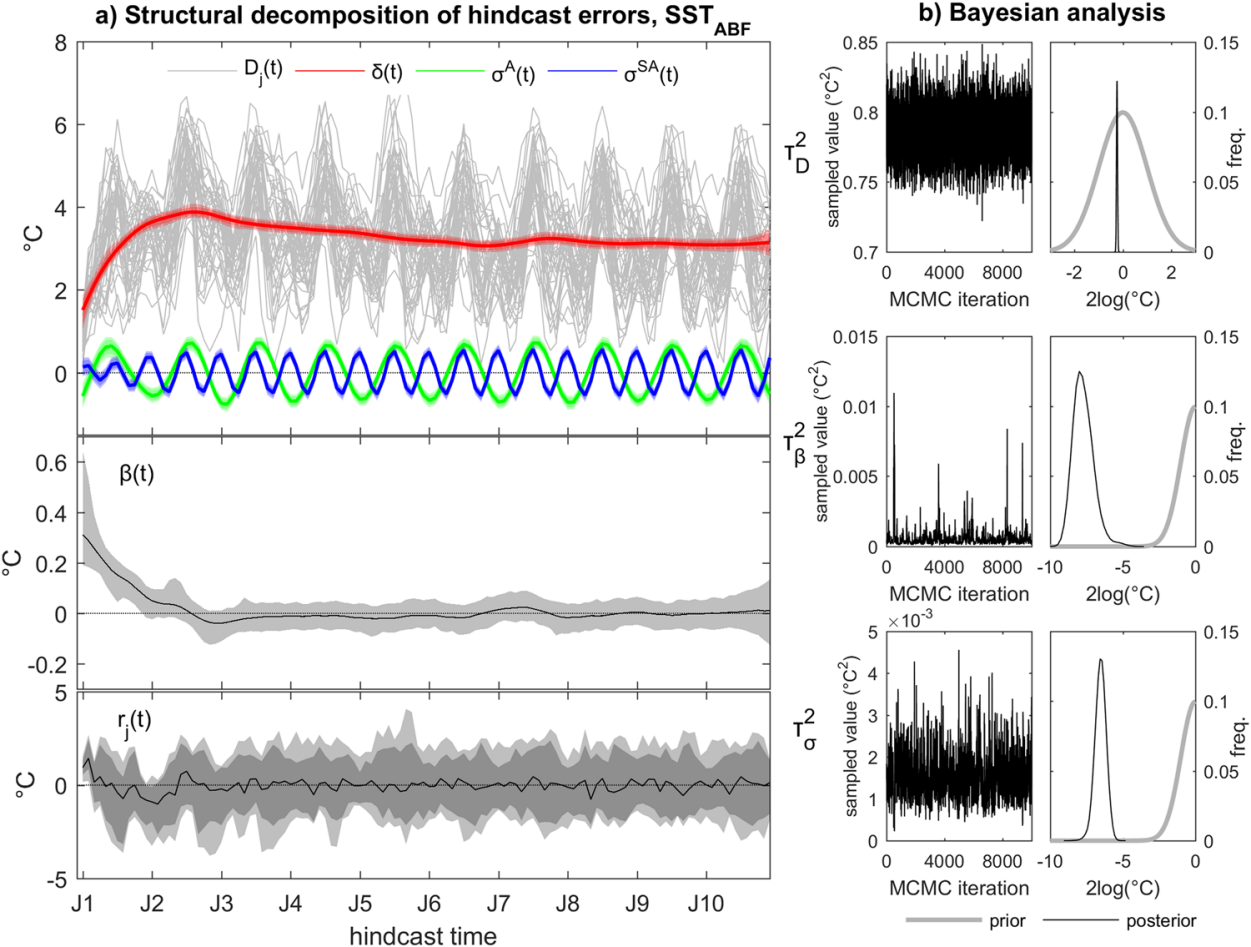
Structural decomposition and Bayesian analysis of decadal climate prediction errors. (**a**) (Top panel) grey lines: empirical hindcast errors in monthly mean spatially-averaged SST in the Angola-Benguela front region; colored lines/shading: associated posterior marginal estimates of systematic error components including drift/bias (red), annual seasonal bias (green) and semiannual seasonal bias (blue); (mid panel) posterior estimate of the stochastic trend; (bottom panel) posterior residual errors. Lines are posterior marginal medians, dark/light shading are 5^th^–95^th^ and 1^st^–99^th^ percentile ranges. (**b**) (Left) trace plots of the Monte Carlo Markov chains for the three model parameters; (right) posterior (black) versus prior (grey) distributions, shown as logarithms for better visualization. The probability distributions are plotted using 25 bins.

We consider the spatially averaged SST in a region of the south-eastern tropical Atlantic containing the Angola-Benguela frontal zone (SST_ABF_, the region is defined in the methods section). Empirical hindcast errors consist in large seasonal errors superposed on a warm climatological mean bias (grey lines in Fig. 1a). The posterior marginal distributions, i.e., the posterior distributions of the individual systematic error components obtained by the structural decomposition, characterize three major drift phases: an initial strong warming (β(*t*) > 0) in the first two hindcast years, which peaks at ~4 °C; a subsequent progressive weak cooling (β(*t*) < 0), which extends into the 7^th^ hindcast year; and finally a transition into climatological bias conditions (β(*t*) ≈ 0) quantified as a bias of 3.12 [3.00, 3.23] °C – estimated as median and 5^th^–95^th^ percentile range of δ(*t* = *90 …120*). Annual and semiannual seasonal biases – σ^A^(*t*) and σ^SA^(*t*) – with amplitudes of ~1.3 °C and ~1.05 °C, respectively, interfere constructively in July/August and destructively in January/February. Further, the semiannual bias component is substantially damped during the first hindcast year, possibly highlighting the effects of the initial coupling shock. The associated Bayesian analysis (Fig. 1b) demonstrates that the data strongly modify our (weakly informative) prior assumption about the model’s parameters, leading to well constrained posterior estimates. Variance of errors at the data level (τ^2^_D_, see equation (2) of the methods section) is substantially larger than those at the process level, and therefore more strongly contribute to the overall uncertainty in the hindcast errors. Posterior distributions of error variances are noticeably skewed at the process level, particularly for the seasonal bias component (τ^2^_σ_).

**Figure 2:**
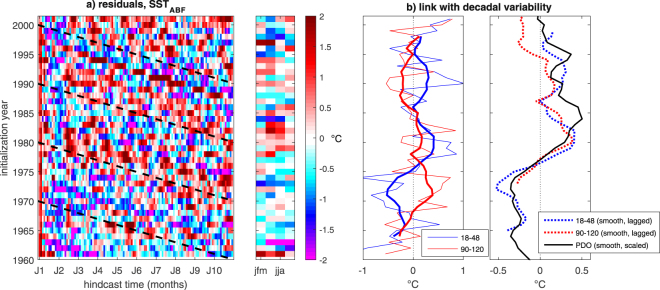
Analysis of the residuals (r_j_(*t*)) of the Bayesian decomposition of monthly mean error signals in spatially-averaged SST in the Angola-Benguela front region. (**a**) Left: posterior median of monthly residuals for each hindcast; black dashed lines indicate the position of selected years along the forecast time axis, and provide a visual guideline to detect systematic drift-corrected error components. Right: seasonal residuals averaged for each simulated year (years refer to different integration years in the different hindcasts). (**b**) Left: temporal evolution of residuals calculated for two different hindcast times (blue: average during hindcast time 18–48 months; red: average during hindcast time 90–120 months); thick lines are 7-year smoothed data (see methods). Right: connection between residuals and the interdecadal internal climate variability expressed by the (7-year smoothed) observed annual-average Pacific Decadal Oscillation (PDO) index. Residuals are 7-year smoothed and lagged by the corresponding average hindcast time (lags are 3 and 9 years for the blue and red curve, respectively).

The residuals r_j_(*t*), i.e., the differences between D_j_(*t*) and the Bayesian estimates of Δ(*t*) (see Fig. 1a, bottom panel), become roughly stationary around zero and homoskedastic (i.e., consistent with being drawn from the same distribution at all time steps *t*) from roughly the third integration year onward, indicating that our decomposition captures the bulk evolution of systematic hindcast errors. However, systematic departures from zero of the residuals are diagnosed in the first two hindcast years. The positive residuals in the first few months and the negative residuals around January of the second integration year possibly reflect unresolved issues with the seasonal error (i.e., the initial shock). Figure 2 expands the residual analysis to the case of individual hindcasts: stripes of similar colors in Fig. 2a that are parallel to the black dashed lines characterize specific events that the decadal climate prediction system systematically fails to predict. Among these events are the El Niños of 1972/73, 1982 (concomitant with the El Chichón eruption), 1991/92 (concomitant with the Pinatubo eruption) and 1997/98. For long hindcast times (>2 years), failure to predict these events is not unexpected since their occurrence is determined by the chaotic nature of the climate system. It also reflects the difficulties inherent to simulating the Atlantic response to the Pacific^26^.

The hindcasts also fail to predict multiannual SST_ABF_ anomalies. The residual error signal displays an interdecadal fluctuation with predominant negative errors before the mid-1970s and predominant warm errors afterwards. This signal robustly emerges in different seasons (Fig. 2a, right panel) and for different hindcast times (Fig. 2b). Accounting for the delay of the error signal with respect to the initialization year, its interdecadal modulation can be shown to match the shift of the Pacific Decadal Oscillation (PDO) from a cold to a warm phase in the mid-1970s (Fig. 2b, right panel).

**Figure 3:**
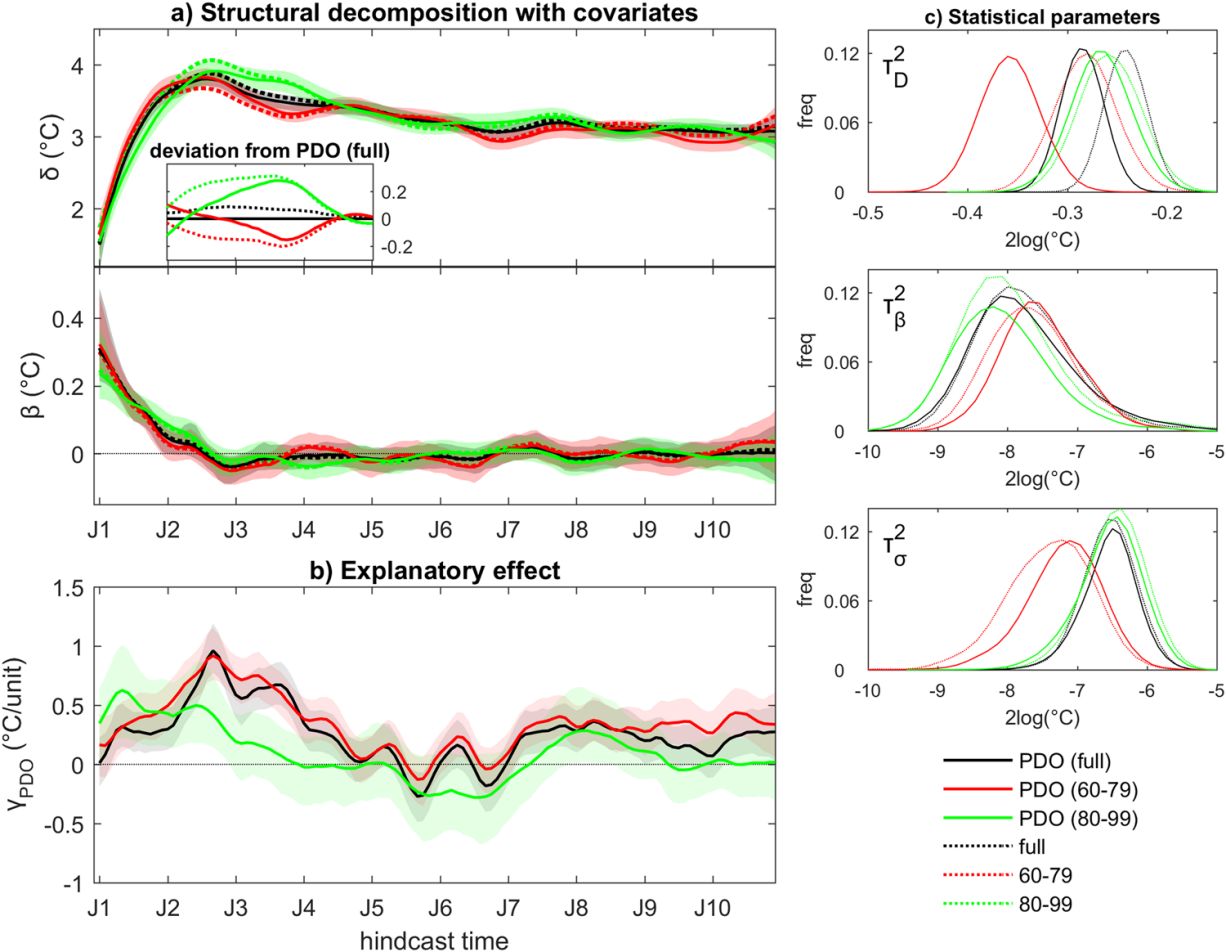
Explanatory effect of unpredicted inter-decadal climate variability on the structural decomposition of decadal climate prediction errors. (**a**) Posterior marginal estimates of drift/bias (δ) and stochastic trend (β) from hindcast errors in monthly mean spatially-averaged SST in the Angola-Benguela front region for three ensembles (full ensemble, sub-ensemble including initialization years from 1960 to 1979, sub-ensemble including initialization years from 1970 to 1999). Results including the observed Pacific Decadal Oscillation (PDO) index as covariate (continuous lines: median; shading: 5^th^–95^th^ percentile range) are compared with corresponding results without any covariate (dashed lines: median). The inset in the top panel illustrates the differences in the posterior medians relative to the estimate for the full-ensemble with PDO for the 13–48 month hindcast period. (**b**) Posterior marginal estimates of regression coefficients γ for the PDO index in the different setups. (**c**) Posterior empirical distributions of the main parameters in the different model setups, shown as logarithm for better visualization. Representation as in Fig. 1.

Based on this empirical analysis, the statistical model for the estimation of systematic SST_ABF_ errors is expanded to assess the explanatory effect of the PDO. In practice, the observed PDO index is included as an additional covariate in the state-space model, and the posterior marginal distributions of all unknowns obtained from the expanded model are compared with those obtained in the original analysis illustrated in Fig. 1. Consistent with the analysis of residuals without co-variates, the PDO index is lagged by 24 months, i.e., X(*t*) = PDO(*t-24*). The approach is applied to the full hindcast ensemble and for two equally-sized sub-ensembles defined by the period of the hindcast initialization year, i.e., 1960–1979 and 1980–1999. In the full-ensemble analysis, the stochastic regression coefficient γ_PDO_(*t*) indicates that the PDO index is informative about SST_ABF_ hindcast errors, with strongest impact in the second and third hindcast years (black line/shading in Fig. 3b). Inclusion of the PDO index does not appreciably affect the posterior estimation of the parameters of the systematic components (posterior estimates as black lines for τ^2^_β_ and τ^2^_σ_ in Fig. 3c), but it substantially reduces uncertainty in the non-systematic component (see τ^2^_D_ in Fig. 3c). Similar damping of τ^2^_D_ is inferred also for the two sub-ensembles. The PDO index is relevant for the estimation of SST_ABF_ hindcast errors for both sub-ensembles, but with a smaller impact in the 1980–1999 sub-ensemble (Fig. 3b), in line with the weaker relationship seen in the empirical analysis (Fig. 2b). The PDO-corrected posterior estimates of δ(*t*) agree with the full-period estimate better than the corresponding estimates from a model without covariates, particularly around the peak warm error phase (compare dashed and continuous lines in Fig. 3a). A plausible interpretation may be given as follows. A teleconnection exists between the PDO and the southeastern tropical Atlantic, which is observed as a negative PDO-SST_ABF_ correlation; the positive explanatory term γ_PDO_ (in this context, a sort of correction term) suggests that this teleconnection is active also in the simulated climate; removing the effects of remote forcing from the erroneous Pacific state leads to improved estimation of the local drift component. In general, it can be concluded that the estimation of the drift and of its uncertainty depends, to some extent, on the systematic portion of hindcast errors that depends on (inter)decadal internal climate variability. Our method could therefore be used in practice to improve drift estimation by accounting for known explanatory factors of such internal climate variability.

**Figure 4:**
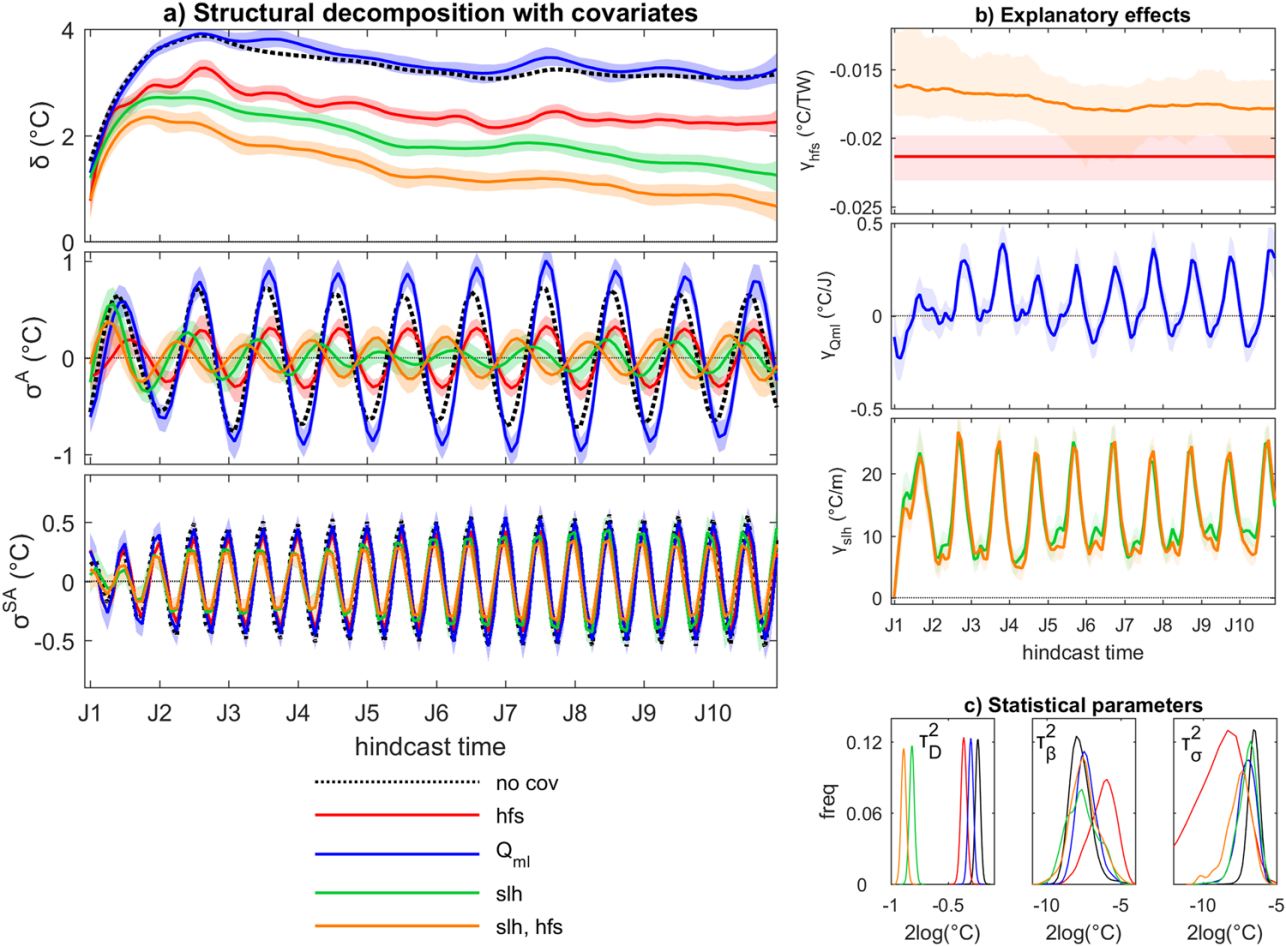
Explanatory effects of covariates on the structural decomposition of decadal climate prediction errors. (**a**) Posterior marginal estimates of systematic error components from hindcast errors in monthly mean spatially-averaged SST in the Angola-Benguela front region, including (from top to bottom) drift/bias δ, stochastic trend β, annual seasonal bias σ^A^ and semiannual seasonal bias σ^SA^. Different choices of covariates are shown: adjusted regional surface heat flux into the ocean (shf, red), mixed layer heat content (Q_ml_, blue), 1-month lagged sea-level height (slh, green), hfs and slh in conjunction (orange). Solid lines: median; shading: 5^th^–95^th^ percentile range. The median estimate without covariate is shown for comparison (dashed black, from Fig. 1). (**b**) Posterior marginal estimates of regression coefficients γ for the two covariates in the different setups. (**c**) Posterior empirical distributions of the main parameters in the different model setups. Representation as in Fig. 1.

As a further example for the possibility of including explanatory covariates in the statistical model, Fig. 4a shows the structural decomposition of systematic SST_ABF_ hindcast errors when the effect of hindcast errors in selected terms of the heat budget for the local mixed layer are accounted for. The blue curves in Fig. 4a correspond to results of a model setup accounting for the explanatory effect of the mixed layer heat content (Q_ml_). Whereas empirical estimates of systematic errors (see methods) in Q_ml_ and SST_ABF_ are significantly correlated (r = −0.66, p < 0.001), the Bayesian analysis indicates that systematic SST_ABF_ errors are largely unexplained by Q_ml_ alone, reflective of the weaker average correlation between errors of both variables in the individual hindcasts (r = −0.32) and suggesting a more complex causal association beyond the thermal state of the mixed layer.

Hindcast errors in both, adjusted surface heat flux (hfs) and 1-month lagged sea-level height (slh) reduce the error component δ(*t*) and damp the estimated annual and semiannual biases (red and green lines in Fig. 4a, respectively). Changes in slh tend to reflect ocean currents. Horizontal circulation changes tend to be associated with meridional shifts of the Angola-Benguela SST front, while changes in upwelling result in local shoaling or sinking of the thermocline. With slh as covariate, the annual seasonal bias σ^A^(*t*) nearly vanishes from the second hindcast year onward, and the semiannual seasonal bias σ^SA^(*t*) is also appreciably reduced. The larger amplitudes of σ^A^(*t*) during the first years suggest decoupling of surface from subsurface errors during the initial shock phase. The posterior regression coefficient γ(*t*) (Fig. 4b) corresponding to this parameter illustrates a seasonally-varying connection between errors in SST that is strongest during austral spring, possibly reflecting the seasonality of wind-dependent errors. Analysis of the posterior marginal distributions of the statistical model parameters indicates that inclusion of slh as explanatory covariate largely damps the variance of the observational error (Fig. 4c), which is the major source of uncertainty in the original estimation (Fig. 1b).

An even larger reduction of δ(*t*) is obtained when the explanatory effects of hindcast errors in hfs and slh are accounted for in conjunction (orange lines in Fig. 4a). With this choice, δ(*t*) has values below 1 °C for long hindcast times, indicating that the climatological bias is almost completely damped when accounting for the explanatory effects of hfs and slh. The posterior estimate of σ^A^(*t*) obtained with this model configuration is phase-shifted compared to the original analysis and has larger amplitudes compared to the configuration with slh as the only covariate, which suggests concomitant effects on SST biases from misrepresented seasonal variations in hfs and slh that mutually compensate or reinforce each other depending on their phase.

The SST_ABF_ drift thus largely develops due to erroneous heat gain by the ocean through surface fluxes coupled with an erroneous redistribution of that heat in the water mass. In contrast, an only marginal connection is found with associated systematic errors in the mixed layer heat content, which are in turn dominated by the erroneous volume loss simulated in the mixed layer. Overall, the analysis highlights the complexity of errors in a region featuring a great variety of coupled dynamics and teleconnections^27^. Fully disentangling such complexity exceeds the scope of the illustrative analysis presented here.

Figures 1c, 3c and 4c consistently show that the largest uncertainty that affects the estimation of the SST_ABF_ drift stems from the data model parameter τ^2^_D_ included in the observation equation of our statistical model (see the methods section). We tested the impact of using different input data to the dynamic linear model by constructing different hindcast ensembles, from small sub-ensembles to that used in the main analysis to super-ensembles including multiple realizations for each hindcast (results not shown). Tighter constrains on τ^2^_D_ are generally achieved by increasing the size of the ensemble, without significant impacts on its estimated mean. By contrast, reducing the ensemble size can lead to major discrepancies in the mean estimation. It is especially in this case that the proposed Bayesian hierarchical formulation could be valuable – through the implementation of prior knowledge about uncertainty parameters and/or explanatory factors, as shown in Fig. 3 – to partly overcome the dependency of drift uncertainty estimation on the quality and quantity of available empirical information about the drift.

**Figure 5:**
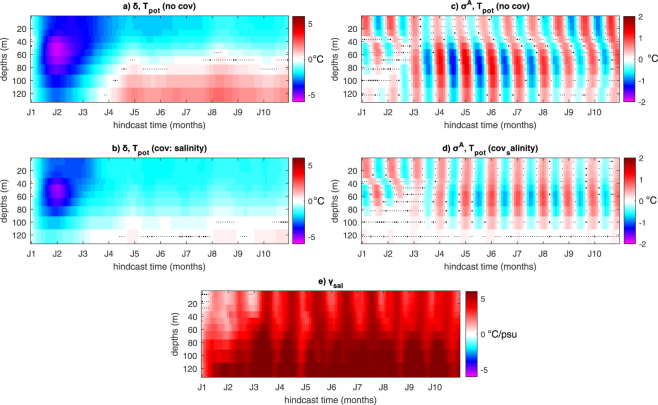
Evolution of systematic errors (level error δ and annual bias component σ^A^) in seawater potential temperature at 44°S latitude and 50°W longitude for the upper ten model levels. (**a**,**c**) Standalone analysis; (**b**,**d**,**e**) analysis including the explanatory effect of local salinity errors. Posterior estimates were interpolated on a regular longitude-latitude grid before the analysis. Shading: median of posterior marginal errors. Large (small) dots indicate grid points where the estimated value is not significantly different from zero with high (low) confidence, i.e., where the distribution of values crosses the value of zero in between the 40^th^–60^th^ (5^th^–95^th^) percentile range. Posterior estimates were interpolated on a regular longitude-latitude grid before the analysis.

So far, the state-space decomposition method has been illustrated for a single predictand, SST_ABF_. Its analytic potential, however, is exploited more fully when it is applied simultaneously to spatially distributed data. Here, we illustrate how it may aid the characterization of the spatio-temporal complexity of decadal climate prediction errors, and assist in understanding the underlying dynamics with an application to gridded upper-ocean potential temperature data in the southern Atlantic mid-latitudes, near the Brazil–Malvinas confluence zone. The results indicate intricate variability in the sub-surface ocean for the δ(*t*) and σ^A^(*t*) components (see supplementary movie). For instance, at [44°S, 50°W] during the initial drift phase negative (i.e., cold) errors (δ(*t*) < 0) are diagnosed throughout the water column down to 120 m depth, with peak negative values around 50 m depth, while errors of opposite sign characterize near-surface and subsurface evolution during the climatological bias phase (Fig. 5a). In the annual bias component, downwelling pulses of both warm and cold errors are observed (see Fig. 5c). Inclusion of hindcast errors in local seawater salinity as covariate in the state-space model indicates that a considerable part of potential temperature errors in the subsurface ocean is linked with concomitant errors in salinity. Following the initial drift phase, the warm climatological bias below 80 m depth is largely damped in the model including salinity errors as covariate (compare panels a and b of Fig. 5). Also, annual bias fluctuations are damped in the sub-surface ocean while they are phase-shifted in the near-surface layer (compare panels c and d of Fig. 5), as highlighted by the strong seasonal variations of the regression coefficient γ_sal_(*t*) in the near-surface layers (Fig. 5e). Thus, coherent seasonal error evolutions are detected throughout the upper ocean column (Fig. 5d). Overall, the analysis highlights once more how our statistical model aids the identification and quantification of linkages between drift and biases affecting different covarying processes. Specifically, it links downwelling of systematic warm (i.e., buoyancy positive) hindcast errors with overcompensation by corresponding local salinity errors.

## Discussion and Conclusions

We propose a Bayesian hierarchical framework for the unified statistical assessment of systematic hindcast errors in decadal climate predictions. The major novelty of our approach is that the proposed state-space model allows for an explicit statistical estimation of the temporal evolution of major systematic error components, including drift, climatological bias and seasonal biases. It also allows to quantify the explanatory effect of co-varying processes, and to separately evaluate the associated uncertainties at the data, process and parameter levels.

We present an illustrative application of the model for spatially-averaged SST in the Angola-Benguela front region – where coupled climate models are typically affected by a strong warm bias^5^ – simulated by an ensemble modelling system intended for decadal climate predictions. Different aspects of drift/bias quantification and interpretation are discussed, and we demonstrate the value of the proposed statistical approach as a diagnostic, exploratory and hypothesis-testing tool.

**Figure 6:**
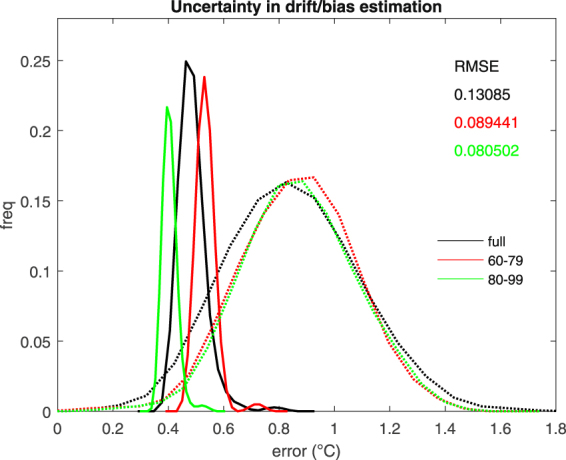
Comparison between the empirical distribution (dashed line) and Bayesian posterior distribution (solid line) of systematic decadal climate prediction errors. Results refer to the monthly mean spatially-averaged SST in the Angola-Benguela front region for the three ensembles referring to different initialization periods used in Fig. 3. Black: full ensemble; red: sub-ensemble including initialization years from 1960 to 1979; green: sub-ensemble including initialization years from 1980 to 1999). Analysis is for drift plus seasonal biases (i.e., δ + σ^A^ + σ^SA^, see equation (3) in the methods section). The shown error distributions are obtained from the monthly estimates calculated over all forecast lead times. These are the 1^st^–99^th^ percentile ranges of the posterior samples for the BHM, and the unbiased sample standard deviation for the empirical method. The probability distributions are plotted using 13 bins. Numbers are the root mean squared errors (RMSEs) between the mean Bayesian and mean empirical estimates of the drift.

First, we show that by virtue of the separation between data and process levels, the hierarchical method allows for well-constrained estimates of statistical uncertainty for the different systematic error components (Fig. 1). Compared to currently adopted empirical approaches, the Bayesian estimates of drift uncertainty yield narrower distributions (Fig. 6). The more reliable estimation of drift uncertainty – at the process level – resulting from this rigorous methodology can provide more robust estimates of prediction skills compared to traditional simple averages. In particular, the posterior ensemble realizations of the drift (and of other systematic hindcast error components) obtained from the Bayesian hierarchical model gives a forecast drift correction analogous to the empirical estimate used in current approaches^28^.

Second, we show that drift estimation is appreciably affected by the interdecadal climate state and evolution, including extant global teleconnections between hindcast errors (Fig. 2). The shorter the analysis period, the larger the risk of erroneously attributing to drift a portion of (inter)decadal climate variability which the system systematically fails to predict. We show that this issue can be in principle overcome within our statistical modeling framework by accounting for the explanatory effect of such unresolved (inter)decadal climate variability (Fig. 3).

Finally, we show that the proposed structural decomposition permits a robust quantification of non-stationary explanatory effects of the bias and their uncertainty (Figs 4 and 5), leading to the possibility of probing causal hypotheses for the origin of model biases with a statistically rigorous quantification that is inaccessible to commonly used approaches in traditional multivariate assessments of systematic decadal climate prediction errors. We stress that the proposed hierarchical framework uniquely allows for a fully traceable quantification of explanatory effects based on the interdependencies expressed in each individual hindcast.

We believe that the strength of these advantages, together with their modest computational requirements, justify the view that state-space models like the one proposed here, or variants of greater complexity (for instance accounting for spatial dependencies^29^, can be used in future applications to robustly and quantitatively assess explanatory effects and uncertainties in climate forecasts. Ultimately, improved drift adjustment techniques such as that outlined here will help to advance the quality of the estimation of the predictability and the predictive skills of a forecast system^20^.

To conclude, the generality of our results make them relevant well beyond the specific decadal prediction system, the focus region or reference variable employed in this study. Compared with the existing empirical techniques for drift estimation, the structural decomposition and Bayesian hierarchical approach envisaged here represent an opportunity for progress in our understanding of systematic climate model errors: the method yields a reliable estimation of drift and bias within a unified framework; it permits an evaluation of causal relationships and teleconnections by embedding relevant information on associated dynamics at the process level; and it allows to make a quantitative separation of the process-related uncertainties from those associated to empirical data limitations.

## Methods

### Bayesian approach

Scientific literature contains numerous studies^29–34^ describing the application of Bayesian hierarchical models (BHMs) in the field of climate research, especially to the assessment of climate model outputs. BHMs use Bayes’ theorem to incorporate information from different sources, including observations, physical theories and experts’ knowledge. They provide a flexible framework to developing consistent inference and prediction of unknown quantities under study, which overcomes single-value estimates of uncertainty.

The BHM general formulation is based on three building blocks: the data model, the process model and the parameter model. Suppose that ***Z*** represents the data, ***Y*** represents the process and ***θ*** represents the parameters related to the data and the process model, we then have:

- Data model, [***Z***|***Y***, ***θ***]
- Process model, [***Y***|***θ***]
- Parameter model, [***θ***]

where [A] is the generic notation for the probability distribution of the random quantity A. In practice, (a) defines the statistical model representing the dependence of observations on the unknown process, (b) describes the conditional probability distribution of the process on the model parameters, and (c) describes the (prior) probability distribution of the parameters, which are treated as random quantities according to the Bayesian approach.

BHMs provide estimates of the unknowns (***Y*** and ***θ***) with associated uncertainty through the calculation of their posterior distribution conditioned to available observations, which is allowed by the Bayes’ theorem:

1 $$[{\boldsymbol{Y}},{\boldsymbol{\theta }}|{\boldsymbol{Z}}]=[{\boldsymbol{Z}}|{\boldsymbol{Y}}][{\boldsymbol{Y}}|{\boldsymbol{\theta }}][{\boldsymbol{\theta }}]/[{\boldsymbol{Z}}]$$

Direct evaluation of (1) to obtain the posterior distribution on the left term is often computationally intractable. This can be circumvented by generating samples from the posterior distribution through a Markov Chain Monte Carlo method^35^.

### Statistical model for the estimation of systematic decadal climate prediction errors

At the data level, the systematic error Δ(*t*) of the decadal climate prediction system at prediction time *t* is observed through differences between the predicted and the observed – or, analogously, assimilated – values (D_j_(*t*)) from an ensemble of *j* = 1,…,p hindcasts initialized at different times, according to the following model:

2 $${{\rm{D}}}_{{\rm{j}}}(t)={\rm{\Delta }}(t)+{{\rm{\varepsilon }}}_{{\rm{j}}}(t)$$

where ε_j_(*t*) is a Gaussian white noise random error with zero mean and variance τ^2^_D_. We assume, for simplicity, that the observation error has the same variance in all hindcasts.

At the process level, Δ(*t*) is decomposed into two components: the drift/bias δ(*t*) and the seasonal bias σ(*t*), further split into annual (σ^A^(*t*)) and semiannual (σ^SA^(*t*)). Namely:

3 $${\rm{\Delta }}(t)={\rm{\delta }}(t)+{{\rm{\sigma }}}^{{\rm{A}}}(t)+{{\rm{\sigma }}}^{{\rm{SA}}}(t)$$

The drift/bias δ(*t*) changes through time according to a local linear trend:

4 $${\rm{\delta }}(t)={\rm{\delta }}(t-{1})+{\rm{\beta }}(t-{1})+{{\rm{\varepsilon }}}_{{\rm{\delta }}}(t)$$

5 $${\rm{\beta }}(t)={\rm{\beta }}(t-{1})+{{\rm{\varepsilon }}}_{{\rm{\beta }}}(t)$$

where ε_δ_(*t*) and ε_β_(*t*) are uncorrelated Gaussian white noise random errors with zero mean and variance τ^2^_δ_ and τ^2^_β_, respectively. The term β(t) is a random walk. The effect of ε_δ_(*t*) is to allow the level of the drift/bias to shift up and down, while ε_β_(*t*) allows the slope to change. The larger the variances, the greater the stochastic movements in the drift/bias. If τ^2^_δ_ = τ^2^_β_ = 0, the local linear trend collapses to a linear deterministic trend.

Seasonal terms are modeled using harmonic functions^36^. Using monthly data with *k* = 1 for σ^A^(*t*) and *k* = 2 for σ^SA^(*t*), the *k* ^th^ harmonic function takes the general form

$${{\rm{\sigma }}}_{{\rm{k}}}(t)={{\zeta }^{1}}_{{\rm{k}}}\,\cos (2{\rm{\pi }}kt/12)+{{\zeta }^{2}}_{{\rm{k}}}\,\sin (2{\rm{\pi }}kt/12)$$

where ζ^1^_*k*_ and ζ^2^_*k*_ are constants. Like the local linear trend, the seasonal term can be built up recursively, leading to the (stochastic) model^36^ for both components:

6 $$(\begin{array}{c}{\sigma }_{{\rm{k}}}(t)\\ {\sigma }_{{\rm{k}}}^{\ast }(t)\end{array})=(\begin{array}{cc}\cos (\mathrm{k2}\pi /\mathrm{12}) & \sin (\mathrm{k2}\pi /\mathrm{12})\\ -\sin (\mathrm{k2}\pi /\mathrm{12}) & \cos (\mathrm{k2}\pi /\mathrm{12})\end{array})(\begin{array}{c}{\sigma }_{{\rm{k}}}(t-1)\\ {\sigma }_{{\rm{k}}}^{\ast }(t-1)\end{array})+(\begin{array}{c}{{\rm{\varepsilon }}}_{\sigma ,k}(t)\\ {{\rm{\varepsilon }}}_{\sigma ,k}^{\ast }(t)\end{array}){\rm{k}}=1,2$$

with ε_*σ*,k_(*t*) and ε*_σ,k_(*t*) uncorrelated Gaussian noise random errors with zero mean and variance τ^2^_*σ*_, and * indicating the conjugate.

The process model (3) can be easily extended to include the effect of external factors in terms of additional explanatory variables. For one dimensional explanatory variable X(*t*), the model becomes:

7 $${\rm{\Delta }}(t)={\rm{\delta }}(t)+{{\rm{\sigma }}}^{{\rm{A}}}(t)+{{\rm{\sigma }}}^{{\rm{SA}}}(t)+{\rm{\gamma }}(t){\rm{X}}(t)$$

In equation (7) we allow γ(*t*) to vary according to a random walk:

8 $${\rm{\gamma }}(t)={\rm{\gamma }}(t-{1})+{{\rm{\varepsilon }}}_{{\rm{\gamma }}}(t)$$

with ε_γ_(*t*) Gaussian white noises with zero mean and variance τ^2^_γ_. Time varying coeffcients γ(*t*) allow us to take into account possible non stationary effects of the covariate. Again, if τ^2^_γ_ = 0, the effect collapses to be constant in time. Finally, the parameter level requires the specification of the prior distribution for the unknown parameters ***θ*** = (τ^2^_D_, τ^2^_δ_, τ^2^_β_, τ^2^_σ_, τ^2^_γ_). In this study, we set τ^2^_δ_ = 0 to obtain a smoothly varying error δ(*t*), which then better corresponds to the drift/bias. We define weakly informative lognormal (LN) priors in the form LN(0,1) for all parameters.

### Dynamic Linear Model implementation

The formulation of the statistical model above allows for a straightforward implementation within the dynamic linear model (DLM) framework. DLMs are based on a state-space approach, i.e., unobservable state variables are used that allow direct modeling of the process (***Y***) generating the observed data (***Z***)^36,37^. DLMs have the general form:

9 $${\boldsymbol{Z}}(t)={\boldsymbol{FY}}(t)+{\bf{v}}(t)$$

10 $${\boldsymbol{Y}}(t)={\boldsymbol{GY}}(t-{1})+{\bf{w}}(t)$$

where *t* is the discrete time variable representing, in our case, monthly values, ***Z***(*t*) is a vector of *p* observations at time *t*, ***Y***(*t*) is the underlying state vector of dimension *m*, **G** is the *m*x*m* system matrix, and **F** is the *m*x*p* observation matrix. We suppose that **v**(*t*)~N(0,**V**) and **w**(*t*)~N(0,**W**) are the observation and model Gaussian errors, respectively, that are serially and mutually uncorrelated. In this formulation, the matrices **V** and **W** contain the model parameters ***θ***. If we suppose that the unknown parameters are random the DLM formulation is a BHM where (9) and (10) are typically referred to as observation equation and system equation, respectively.

We have applied the DLM to spatially-averaged indices and, individually, to the model’s grid-points over the investigated domain. Following (2) and (9), and having *p* values of D(*t*) (i.e., having *p* hindcasts) the observation vector is defined as ***Z***(*t*) = {D^1^(*t*),…, D^p^(*t*)}’.

In the base model without explanatory covariates, following (3) and accounting for annual and semi-annual seasonalities in the decadal climate prediction errors, the state vector is defined as ***Y***(*t*) = {δ(*t*), β(*t*), σ^A^(*t*), σ^A^*(*t*), σ^SA^(*t*), σ^SA^*(*t*)}’. Therefore, the dimension of the state vector is *m* = 6. We further assume **Y**(0)~N(0,0.025), i.e., there is practically no systematic error during the assimilation. The observation matrix **F** is defined following (2) and (3).

The sequential definition of the process model (having a conditional dependency only on the previous time step) allows to use the Kalman filter formulas^36,37^ for calculating the posterior distribution (1) inside a Monte Carlo Markov Chain algorithm. In particular, we use a slice-sampler algorithm^38^ to iteratively sample from the full posterior distribution of the unknown parameters ***θ***.

### Empirical estimation of systematic decadal climate prediction errors

The BHM results are compared with empirical estimates obtained following the guidelines for drift/bias correction by the Decadal Climate Prediction Panel for the upcoming Coupled Model Intercomparison Project phase 6^28^. The associated uncertainty is calculated for each hindcast time *t* as the unbiased sample standard deviation of the D_j_(*t*) values from the *j* = 1,…,p hindcasts. Similar results are obtained using the 95% confidence interval of the standard error of the mean.

### Decadal prediction system and dataset

The MiKlip prototype system for decadal climate predictions^25^ is based on the low-resolution version of the Max Planck Institute - Earth System Model (MPI-ESM-LR). MPI-ESM-LR is a conglomerate of a coupled general circulation model and subsystem models for land and vegetation, and biogeochemistry. In MPI-ESM-LR, the atmospheric general circulation model ECHAM6^39^ uses a T63/1.9° horizontal resolution and 47 hybrid sigma pressure levels that extend up to 0.01hPa; the ocean-sea ice model MPIOM^9^ uses a 1.5° resolution bipolar grid with 40 z-levels. The time step for numerical integration is 600 s for ECHAM6 and 4320 s for MPIOM.

[9] describe the main oceanic biases, highlighting serious biases prevailing in the intermediate layers of the ocean, which reflect the inability of the model to maintain the correct water mass properties. In general, the ocean in MPI-ESM gets too warm and saline at intermediate levels and in the deep ocean whereas it is too cold and fresh in the upper layers. MPI-ESM-LR and similar configurations of MPI-ESM have been widely tested and used in studies of climate dynamics and variability^40,41^.

We use the MiKlip decadal prediction experiments^25^ based on full-field assimilation of the ORAS4 ocean reanalysis data^42^. The lagged initialization procedure is used to obtain the initial state from the historical assimilation run for each of the 15 ensemble members for each initialization year between 1960 and 2000. The external radiative forcing data are based on the recommendations for the Coupled Model Intercomparison Project phase 5 (CMIP5), an overview of which is outlined in^25^. The ensemble including the first ensemble members (“r1”) was used in the main analysis. The associated historical assimilation run is used as analog for the observational targets.

Spatially-averaged indices for the Angola-Benguela front region used in the study refer to the domain spanning 10°S–20°S latitude and 10°E–15°E longitude (to the coastline). Empirical hindcast errors in monthly mean spatially-averaged SST in the Angola-Benguela front region are provided as supplementary information. The observed PDO index is the JISAO PDO index^43^ available at: http://research.jisao.washington.edu/pdo/PDO.latest.txt. The original monthly index was low-pass filtered with an 85-month running average (corresponding to 7 years) before analysis to smooth out ENSO influences. Adjusted surface energy flux for the mixed layer and mixed layer heat content in the ABF region are estimated following^44^. All empirical distributions are smoothed with a 5-point moving average with symmetric Hanning weights.

## Electronic supplementary material

Supplementary Information

Dataset 1

Supplementary Video
