# Supplementary material for: Structural decomposition of decadal climate prediction errors: A Bayesian approach
Source: Sci Rep. 2017 Oct 9;7:12862. doi: 10.1038/s41598-017-13144-2 (PMC5634475; doi:10.1038/s41598-017-13144-2)
Supplement: Supplementary file 1 — Supplementary Information [file 41598_2017_13144_MOESM1_ESM.pdf]

Structural decomposition of decadal climate prediction errors: A Bayesian approach

Zanchettin Davide, Gaetan Carlo, Arisido Maeregu Woldeyes,

Modali Kameswarrao, Toniazzo Thomas, Keenlyside Noel, Rubino Angelo

Supplementary Information

Supplementary Information consists of one video file and one data file:

supplementary_video.avi

Shown is the evolution of selected systematic hindcast error components (level error δ and annual bias component σ^A) in seawater potential temperature (T_{pot}) along a latitudinal section at 44°S latitude between 60°W and 0° longitude for the upper ten model levels. Top panels: standalone analysis (no covariate included); Mid and bottom panels: analysis including the explanatory effect of local salinity errors. Posterior estimates were interpolated on a regular longitude-latitude grid before the analysis. Shading: median of posterior marginal errors. Large (small) dots indicate grid points where the estimated value is not significantly different from zero with high (low) confidence, i.e., where the distribution of values crosses the value of zero in between the 40th-60th (5th-95th)

percentile range. Posterior estimates were interpolated on a regular longitude-latitude grid before the analysis.

DATASET1.xls

Excel file containing the empirical hindcast errors in monthly mean spatially-averaged SST in the Angola-Benguela front region plotted in Figure 1.
